# Use of ultrasound guidance to improve the safety of percutaneous dilatational tracheostomy: a literature review

**DOI:** 10.1186/s13054-015-0942-5

**Published:** 2015-05-18

**Authors:** Mariam Alansari, Hadil Alotair, Zohair Al Aseri, Mohammed A Elhoseny

**Affiliations:** Department of Critical Care Medicine, King Khalid University Hospital, College of Medicine, King Saud University, PO Box: 2925 (95), Riyadh, 11461 Kingdom of Saudi Arabia; Department of Emergency Medicine, King Khalid University Hospital, College of Medicine, King Saud University, PO Box: 2925 (95), Riyadh, 11461 Kingdom of Saudi Arabia

## Abstract

Patients in ICUs frequently require tracheostomy for long-term ventilator support, and the percutaneous dilatational tracheostomy (PDT) method is preferred over surgical tracheostomy. The use of ultrasound (US) imaging to guide ICU procedures and interventions has recently emerged as a simple and noninvasive tool. The current evidence regarding the applications of US in PDT is encouraging; however, the exact role of US-guided dilatational tracheostomy (US-PDT) remains poorly defined. In this review, we describe the best available evidence concerning the safety and efficacy of US-PDT and briefly delineate the general principles of US image generation for the airway and of US-PDT procedures.

## Introduction

The remarkable progress in the field of imaging has revolutionised the approach to critically ill patients. Advances in medical technology have made ultrasound (US) imaging less costly, easier to use, more precise, and sufficiently portable to be truly hand-carried. In ICUs, ultrasound scanning (USS) is frequently used to guide vascular access and procedures and for pleural and cardiac imaging. The use of USS in percutaneous dilatational tracheostomy (PDT) has the potential to improve the efficacy and reduce the complications of this common ICU procedure.

## Background

PDT is commonly performed at the bedside in the ICU [1]. With an expected increase in the number of mechanically ventilated patients, a further increase in the number of PDT procedures performed in the ICU is also expected [2, 3]. Despite the frequency with which it is used, PDT does have limitations and risks. It is associated with higher incidences of decannulation and obstruction, particularly when it is performed by an inexperienced operator [4, 5]. Although the overall PDT complication rate is low, serious adverse events, including death, have been reported. In a national survey distributed to Head and Neck Surgery members in the American Academy of Otolaryngology via the academy’s weekly email newsletter (during April and May 2011), information about catastrophic complications during and after tracheotomy was collected. Approximately 500 patients have died or been permanently disabled because of tracheotomy [6]. According to Simon and colleagues [7], a PDT-related death occurs in 1 out of every 600 patients who undergo PDT. Thirty-one percent of these deaths occur during the procedure, secondary to PDT-related haemorrhage. The second most frequent cause of death was airway complications (29.6 %). In this report, bronchoscope guidance was used in 46.5 % of cases, but neither pre-nor intra-procedural PDT US was performed. In another study, most of the serious bleeding incidents were related to unrecognised and unanticipated anatomical variations in the vascular anatomy [8].

Bronchoscope guidance during PDT is used by many institutions as an additional safety adjunct [9, 10]. It enables the confirmation of midline puncture of the trachea and allows the visualisation of posterior tracheal wall injuries, but it is not without complications [11–16]. Therefore, critical care physicians are urged to consider using other tools that could provide a better understanding of the anatomy of the neck to guide PDT procedures and improve their safety. The use of USS before, during, and after PDT could play a role in landmarking, identifying vulnerable structures, choosing the appropriate tracheostomy size, and providing real-time guidance for needle penetration. The scarce number of reports on the use of US-guided PDT (US-PDT) and its recent inclusion in the Australian and New Zealand Intensive Care Society practice guidelines [17] mandate a systematic review of this procedure. We performed such a review to evaluate the available literature that examined the safety and efficacy of US-PDT compared with standard techniques.

## Literature and evidence for the use of ultrasound guided-percutaneous dilatational tracheostomy

### Methods

We performed a literature review to examine the efficacy and safety of US before or during PDT or both. MEDLINE, PubMed, EMBASE, and the Cochrane Central Register of Controlled Trials were searched for trials reporting on the safety and efficacy of US or bronchoscope guidance before and during PDT. The terms used were ‘safety efficacy’, ‘ultrasound’, ‘tracheostomy’, and ‘tracheotomy’.

Prospective trials that reported procedural safety or efficacy data for both the intervention group (US-PDT) and a control group of standard or bronchoscope-guided PDT cases were included. Case reports, review articles, letters to the editor, and surveys were excluded.

### Results

The following sections summarise the role of USS use before, during, and after PDT.

#### Landmarking

The difficulties encountered in locating anatomical landmarks, such as the crico-thyroid membrane, and difficulties with tracheal puncture when the tracheal anatomy is not readily palpable are well documented [18]. Consequently, performing PDT in obese patients and patients with anatomical deformities of the neck carries a high risk of procedural complications [19]. USS of the upper airway can provide important anatomical information that would not be evident upon clinical examination alone, including information about the anatomy of the pre- and paratracheal region. USS is a safe, rapid, repeatable, portable, and widely available tool that performs well in both simulated environments and clinical practice [20–23].

#### Choosing an appropriate tracheostomy size

US-PDT can be used to measure the distance from the skin to the trachea, thus allowing the selection of an appropriately sized tracheostomy tube (that is, regular or extended length) [24].

#### Identifying vulnerable structures

Another advantage of pre-procedure USS of the neck is that it allows the identification of vulnerable structures, such as blood vessels and the thyroid gland, in the neck prior to PDT. Moreover, real-time USS performed during the procedure plays a major role in revealing potentially aberrant vessels, which allows needles and dilators to be guided away from at-risk structures, thus avoiding immediate vascular complications [8]. Several studies of fatal bleeding following PDT revealed that the addition of an ultrasonographic examination to determine the level for the PDT can help operators avoid blood vessels and visualise unanticipated anatomical variations, thereby diminishing the risk of major bleeding [22, 25, 26].

#### Choosing an adequate puncture location (inter-tracheal space and midline)

The routine use of USS also enables the clear visualisation of the tracheal rings, which is necessary for the appropriate positioning of the tracheal puncture and correct midline placement [27–30]. Chacko and colleagues [31] used USS to identify the desired level of tracheal puncture on the transverse axis at the midline. This procedure was guided by pointing the needle tip toward the midline. These authors reported a median time to guide-wire insertion of 12 seconds, and the completion of the entire procedure required 12 minutes [31]. Similarly, Sustić and colleagues [32] reported that the average time required to perform US-PDT was 8 minutes, compared with 12 minutes in the surgical tracheostomy group (*P* < 0.05).

Earlier studies of models have shown that, in subjects with simulated unidentifiable anterior neck airway anatomy and tracheal deviation, the use of USS improved the speed and success of cannula placement and reduced the number of attempts required. Sustić [33] reported that the first-attempt success rate in the correct tracheal puncture site was between 96 and 100 %. No patients in the real-time US group suffered cranial misplacement of the tracheostomy tube, compared with 33 % of the patients in the landmark group (*P* < 0.05) [34]. It is worth mentioning that an increased rate of tracheal stenosis is associated with the proximal placement of the tracheostomy tube between the cricoid cartilage and the first tracheal ring; such a placement is unlikely to occur with the use of US-PDT [35–39].

#### Possible posterior wall tracheal injury (bronchoscope-guided versus ultrasound-guided percutaneous dilatational tracheostomy)

Real-time US guidance makes it possible to follow the needle path during tracheal puncture and to determine the final position of the tracheostomy tube [40, 41]. However, intraluminal air prevents the visualisation of structures such as the posterior pharynx and the posterior wall of the trachea with USS; therefore, injury to the posterior wall of the trachea cannot be completely avoided [42].

Kollig and colleagues [43] reported on 72 consecutive patients requiring PDT who underwent pre-procedure USS evaluation of the neck to assess the level of tracheal puncture, followed by bronchoscope-guided PDT to identify posterior wall injury. Bronchoscope use during the tracheal puncture carries safety risks resulting from the possibility of inaccurate device placement and associated temporary hypoventilation. Moreover, accidental needle puncture of the bronchoscope is a common problem that can lead to high repair costs. The disadvantages of not using a bronchoscope include diminished control of the airway, an inability to detect posterior wall injury and ring fractures, a decreased ability to perform pulmonary toilet around the procedure site, and a diminished ability to detect false passage of the tube, particularly if the guide wire is accidentally pulled back too far.

No studies have yet compared bronchoscope-guided PDT alone with US-guided PDT. Such a comparison is mandatory if we are to improve the PDT technique, minimise patient risks, and eliminate financial burdens.

#### Detecting pneumothorax

The use of portable US to detect pneumothorax has long been studied [44]. Recently, an evidence-based review of the literature compared the abilities of ultrasonography with chest radiography to detect pneumothorax. Bedside ultrasonography was a much more sensitive screening test for pneumothorax compared with supine chest radiography (the time to diagnose pneumothorax was 7 minutes for ultrasonography versus 80 minutes in the x-ray group) [45]. Ultrasonography is an obvious first choice for the post-PDT diagnosis of pneumothorax. However, no studies have yet reported on the detection of post-PDT pneumothorax using ultrasonography.

Only a few prospective randomised controlled trials (RCTs) have compared the safety and efficacy of US-PDT with the traditional landmark-guided technique (with or without bronchoscopy). The remainder of the available studies are all small and largely observational in nature. Table 1 [24, 34, 39, 43, 46–48] summarises the best available evidence to date. Most studies have reported low periprocedural complication rates, but no control groups were used for comparison. It should be noted that the incidence of significant complications following PDT is low; therefore, a large sample size would be required to detect a beneficial effect. No studies have reported long-term follow-up.

**Table 1:** Summary of the best available evidence supporting the use of ultrasound-percutaneous dilatational tracheostomy

Type of US guidance used	Author	Study design	Number of patients	Outcome	Complications
Pre-procedural	Bonde *et al*. [39]	Prospective Observational No control group	28 (excluded patients had severe coagulopathy or were morbidly obese)	Changed puncture location in nine patients (32.1 %); elective vessel ligation in three patients (10.7 %)	Minor bleeding in two patients (7.1 %)
	Kollig *et al*. [43]	Prospective Observational No control group	72	Changed puncture location in 17 patients (23.6 %); changed to surgical tracheostomy in one patient (1.3 %)	Minor bleeding in one patient (1.3 %)
Real-time	Sustić *et al*. [34]	Retrospective Control group was landmark-guided.	26	Cranial misplacement: 0 % versus 33 % (*P* <0.05)	Tracheal ring fracture: 36 % versus 43 % (not significant)
	Rajajee *et al*. [46]	Prospective Feasibility No control group	13	All PDTs were successful. Appropriate positioning of puncture was confirmed on bronchoscopy.	No significant complications were reported.
Pre-procedural US and real-time	Guinot *et al*. [24]	Prospective Observational No control group	50 (obese patients and patients with severe coagulopathy were excluded)	All PDTs were successful. Puncture location was changed in 25 patients (50 %).	Minor bleeding in three patients (6 %); wound infection in one patient (2 %)
Real-time US guidance	Rudas *et al*. [47]	Randomised controlled trial	50	First-pass success rates were 87 % in the US group and 58 % in the landmark group (*P* = 0.028).	The decrease in procedural complications was not statistically significant: 22 % in the US group versus 37 % in the landmark group (*P* = 0.24).
Pre- and post-procedural US	Yavuz *et al*. [48]	Randomised controlled trial	341	The puncture sites designated at the physical examination were reconsidered in 23.8 % of 164 cases. The mean procedure times for the US group and the controls were 24.09 minutes ± 8.05 and 18.62 minutes ± 6.34, respectively (*P* = .001).	The perioperative complication rates were slightly lower in the US group (7.8 %) than in the control group (15.0 %); not statistically significant (*P* = 0.054).

All studies were performed in intensive care units. PDT, percutaneous dilatational tracheostomy; US, ultrasound

Because of the lack of strong supporting evidence, US-PDT is not yet practiced routinely. The small number of available studies suggests that US-PDT is safe and offers a potential benefit over the traditional landmark-guided procedure, particularly in selected patient groups, such as obese patients. Therefore, we decided to describe the procedure in detail (supported by images) to encourage critical care physicians to consider the great safety potential associated with the use of US-PDT and to plan for conducting a large RCT comparing US-PDT with bronchoscope-guided PDT.

## Ultrasound use in percutaneous dilatational tracheostomy

### The basic principle of ultrasound imaging of the airways

Transducer selection is important when evaluating the anatomy of the airway. Higher-frequency linear probes (7.5 MHz) provide better resolution of superficial structures. Therefore, they are the most suitable type of probe for imaging superficial airway structures (within 2 to 3 cm from the skin). In contrast, the curved low-frequency (5 MHz) transducer is best used for obtaining sagittal and parasagittal views of structures in the submandibular and supraglottic regions [49]. These probes produce two-dimensional grayscale images on the screen, ranging from black to white [50].

Hyperechoic structures (such as fat and bone) give a strong echo and thus appear white [51]. Fluid collections or blood vessels let the US beam pass through them easily, creating little echo. They are called anechoic structures, and they appear black on the screen. Cartilaginous structures (such as the thyroid cartilage, the cricoid cartilage, and the tracheal rings) appear as homogeneous, hypoechoic (black) structures. Muscles and connective tissue membranes are also hypoechoic, but they have a more heterogeneous, striated appearance than cartilage does [52]. Air conducts US very weakly, so when the US beam reaches the tissue/air border, a strong reflection (a strong white line) appears, and everything on the screen beyond that point consists of artefacts. Because men have a prominent thyroid cartilage, it is sometimes a challenge to avoid air under the probe when performing a sagittal midline scan from the hyoid bone to the suprasternal notch. This difficulty can be overcome by applying a liberal amount of conductive gel or another fluid-filled interface (for example, a water bath) between the probe and the skin [31, 50].

## The ultrasound-guided percutaneous dilatational tracheostomy procedure and tips

Before proceeding to the PDT, the pre-tracheal area should be examined for the tracheal midline, the tracheal cartilage level, the anterior jugular veins (their diameter and location relative to the midline), and the thyroid isthmus or midline vessel. Aberrant vascular structures crossing the midline can also be observed and should be noted. In evaluating vascular structures, it is important to ensure that the structures are not compressed by excessive pressure from the probe. If in doubt, further evaluation using colour Doppler can be performed [30]. Such pre-procedural USS is likely to lead to a change in the planned location of the tracheal puncture in up to 50 % of cases, mostly to avoid puncturing the thyroid isthmus or an aberrant vessel [43, 53].

Ideally, the space between the first and second or the second and third tracheal rings should be selected for the insertion of the tracheostomy tube. According to sonographic criteria, the point of tracheal puncture should be below the first tracheal ring but above the fifth tracheal ring, with no vascular structures in the path of the needle. The precise inter-tracheal ring space is considered less important than the passage below the first and above the fifth tracheal rings [54].

Avoiding the thyroid isthmus is often not possible and is not believed to be necessary [55]. However, rare but fatal complications, such as arterial bleeding from a thyroid artery or from an avulsed subclavian artery, have been reported during or after tracheostomy. It is also best to avoid making the tracheal access below the third tracheal ring, where the thyroid isthmus is likely to lie [56, 57]. The goal is to puncture the anterior quadrant of the trachea, placing the puncture sites between the 11 and 1 o’clock positions as close as possible to the midline [58].

In morbidly obese patients or patients with anatomical deformities resulting from pathology or injuries, palpation is almost always difficult or even impossible [18, 19, 59]. Therefore, USS should be used in conjunction with palpation of the neck prior to commencing the procedure.

For morbidly obese patients, US should first be used with the head in the neutral position to estimate the thickness of the soft tissue between the skin and the trachea to determine the length of the tracheostomy tube. The mode of imaging should then be adjusted while the trachea is in the centre of the screen to maximise the resolution and depth of imaging.

After induction and positioning, the endotracheal tube should be withdrawn under direct laryngoscopic visualisation until the cuff is positioned immediately inferior to the vocal cords using a standard laryngoscope. Some operators have pulled the endotracheal tube back under US guidance until the cuff is at the level of the thyroid cartilage [60].

**Fig. 1:**
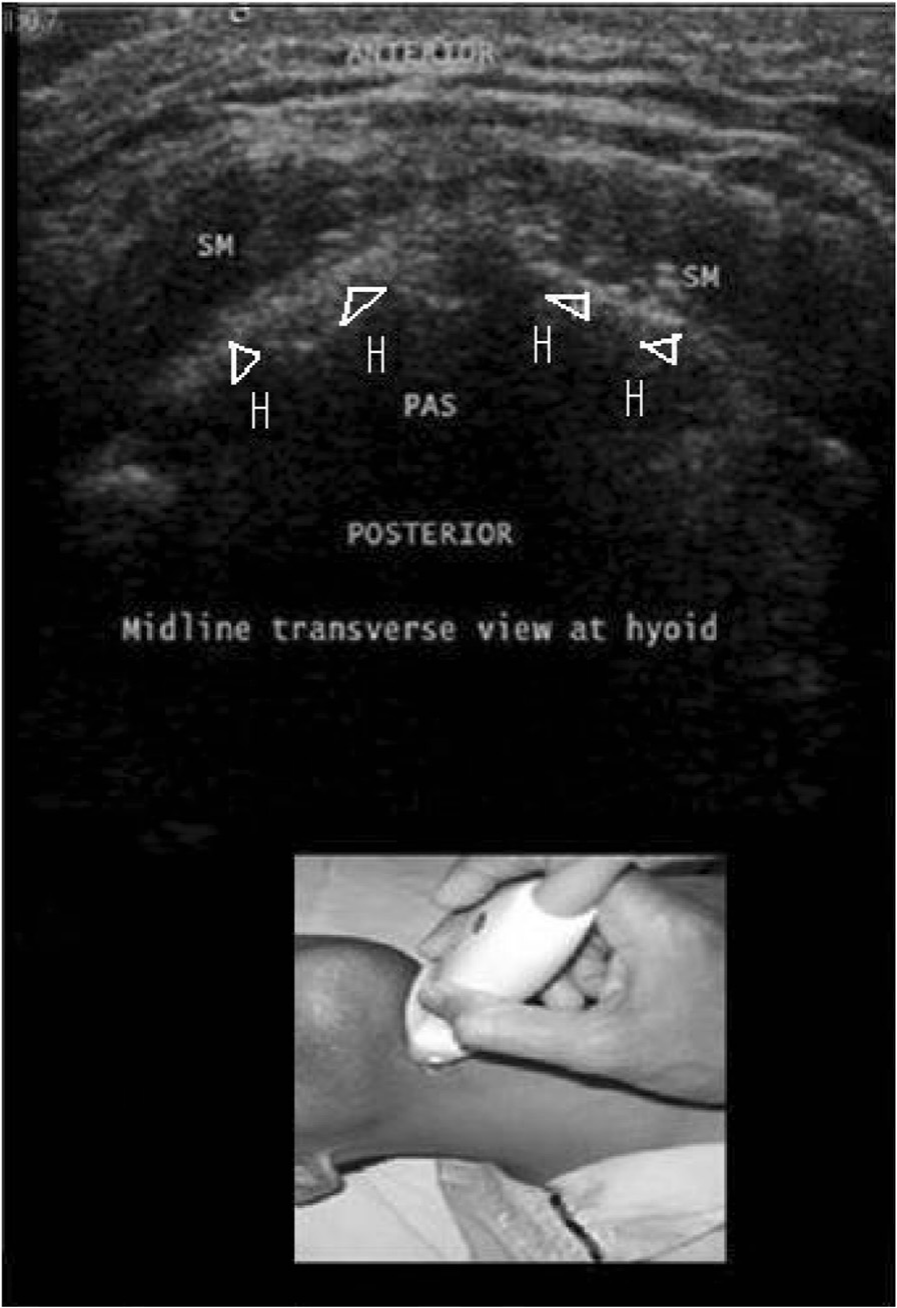
Sonogram of the hyoid bone (H) on a midline transverse view using a linear transducer. The hyoid bone is shown as an inverted, hyper-echoic U. PAS, posterior acoustic shadow; SM, strap muscles. Reprinted with permission from Wolters Kluwer [42]

**Fig. 2:**
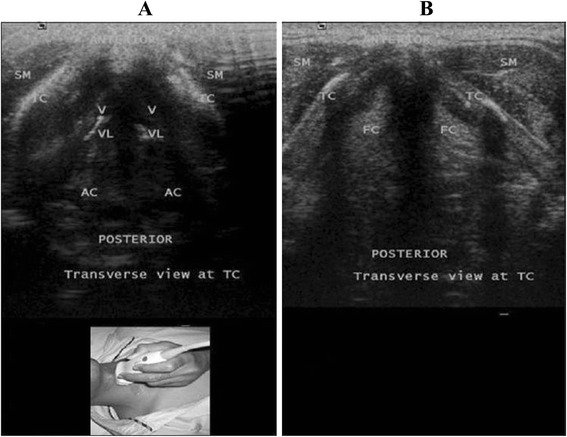
Transverse view at the thyroid cartilage using a linear transducer. Note the difference in echogenicity between the true cords because of the presence of the vocalis muscle **(A)** (TC, Thyroid cartilage; SM, Strap muscles; AC, Arytenoid cartilage; V, Vocalis muscle; VL, Vocal ligaments;) and the increased fat content in the false cords **(B)** (TC, Thyroid cartilage; SM, Strap muscles; FC, False cord;). Reprinted with permission from Wolters Kluwer [42]

With a linear array probe in a sterile sheath, it is possible to obtain transverse/axial real-time images of the upper airway, starting from the hyoid bone down to the thyroid gland and its isthmus. The viewed airway should always be in the centre of the screen. On axial imaging, the airway in the neck should be apparent at the midline and should show mixed hyper-echogenicity. The hyoid bone is a key landmark that separates the upper airway into the suprahyoid and infrahyoid regions (Fig. 1). The inverted V-shaped thyroid cartilage, within which the vocal cords lie, should then be identified caudal to the hyoid bone (Fig. 2).

**Fig. 3:**
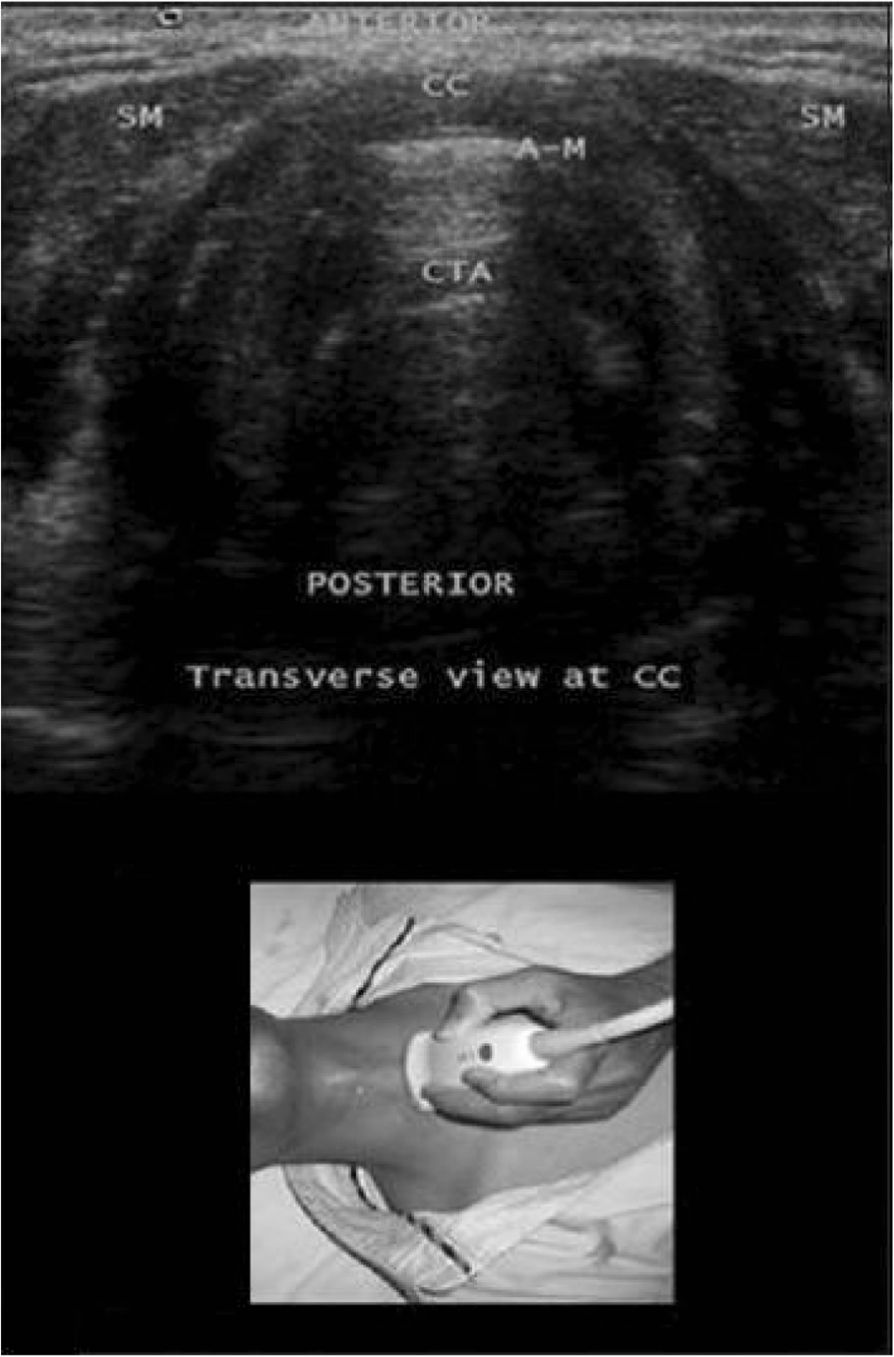
Transverse view at the cricoid cartilage. A-M, air mucosal interface; CC, cricoid cartilage; CTA, comet tail artefacts; SM, strap muscles. Reprinted with permission from Wolters Kluwer [42]

**Fig. 4:**
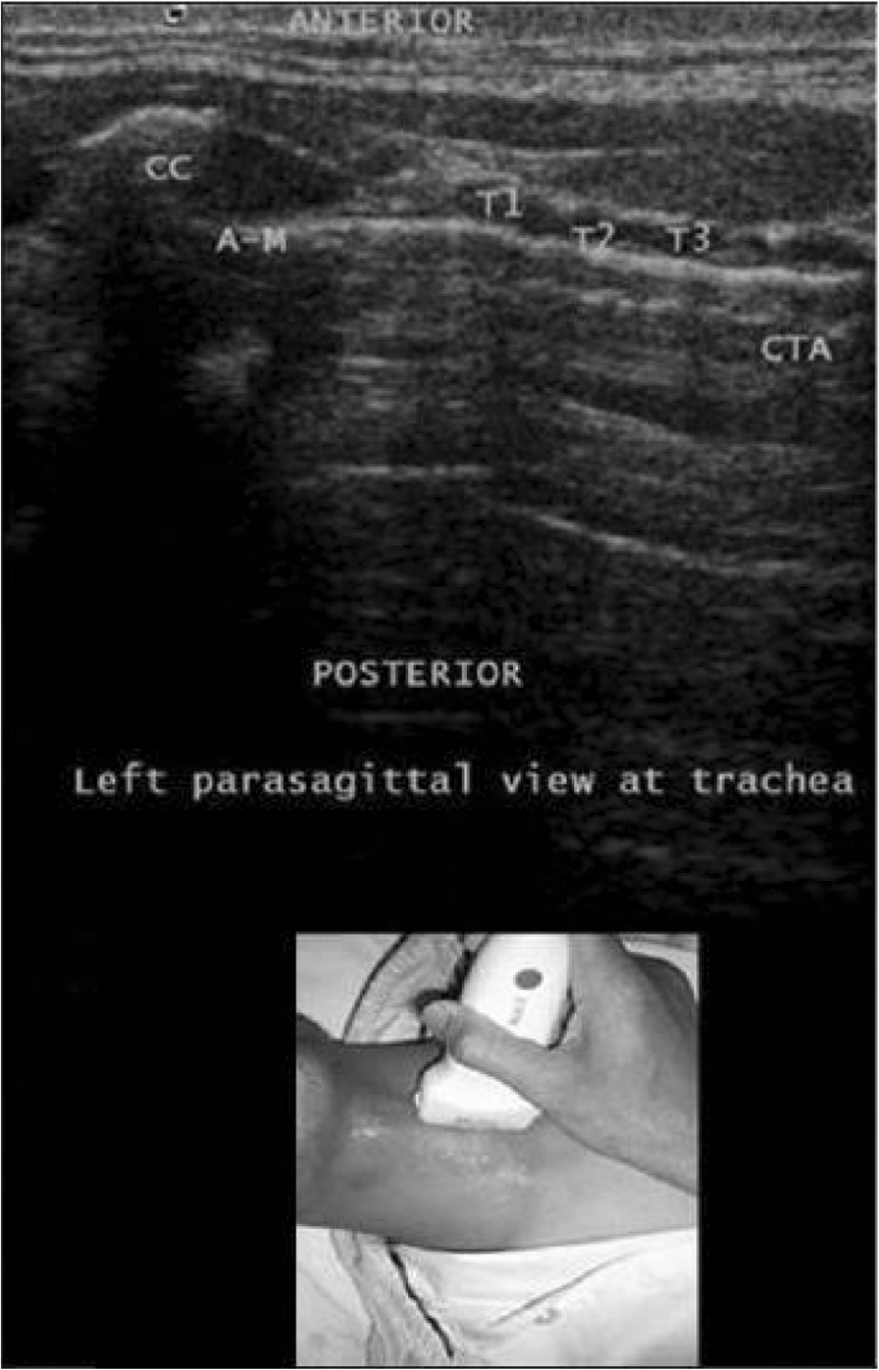
Left parasagittal view at the trachea. A-M, air-mucosal interface; CC, cricoid cartilage; CTA, comet tail artefact; T1-T3, tracheal cartilage. Reprinted with permission from Wolters Kluwer [42]

**Fig. 5:**
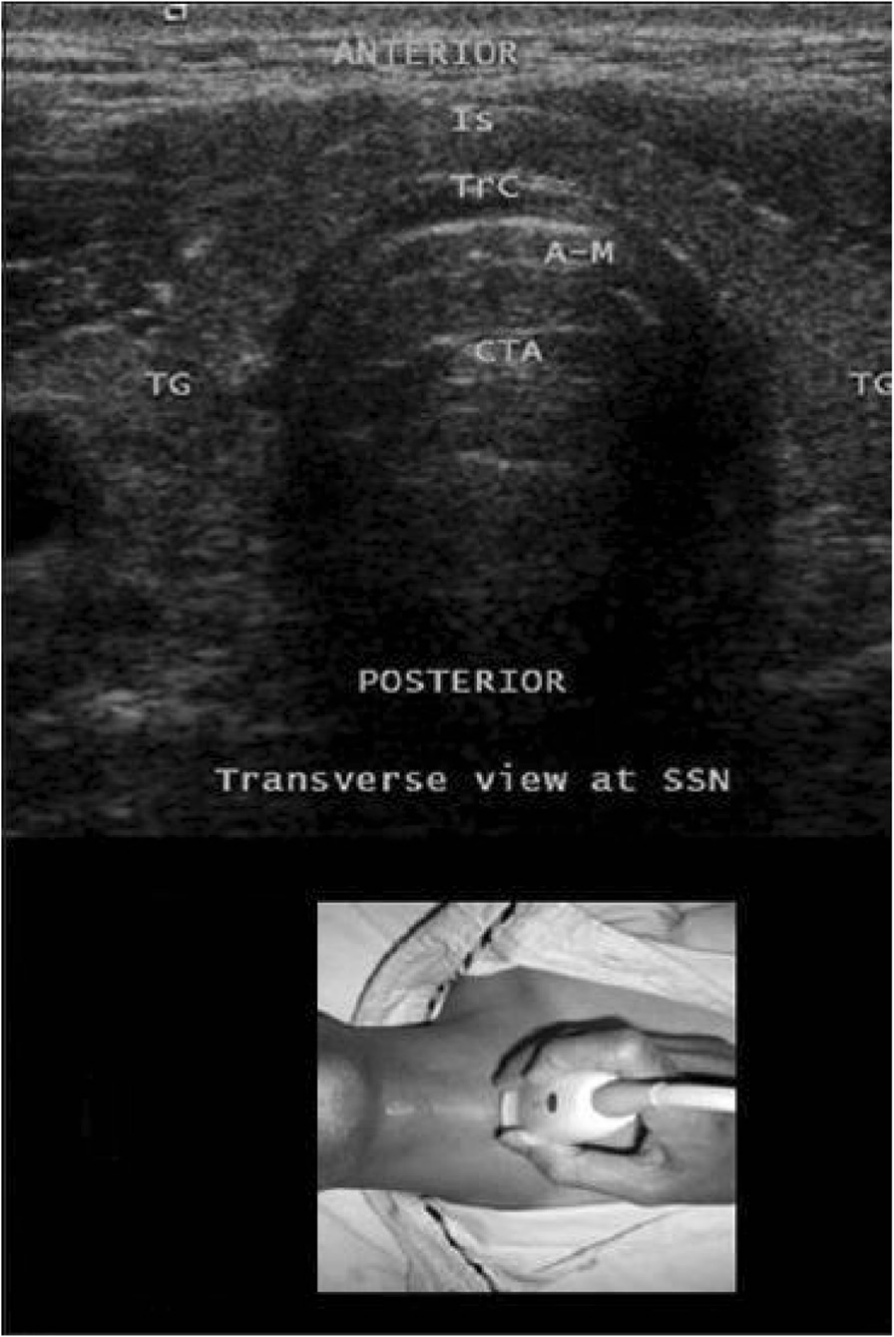
Transverse view at the level of the suprasternal notch (SSN) showing the trachea and the thyroid gland. A-M, air-mucosal interface; CTA, comet tail artefact; Is, isthmus; TG, thyroid gland; Trc, tracheal cartilage (ring). Reprinted with permission from Wolters Kluwer [42]

By sliding the probe caudally, the clinician can identify the cricoid cartilage within the anterior wall of the larynx caudal to the cricothyroid membrane by its relatively large acoustic shadow (seen as a hump on the transverse view; Fig. 3). The posterior surface of its anterior wall is delineated by a bright air-mucosal (A-M) interface and by reverberation artefacts from intraluminal air (Fig. 3). Caudal to the cricoid cartilage, the tracheal rings will be observed. They can be identified by their relatively thin acoustic shadows within the anterior wall of the trachea. Like all cartilaginous structures, the tracheal rings appear hypoechoic. They resemble an inverted U, highlighted by a linear hyper-echoic A-M interface and by reverberation artefacts posteriorly (Fig. 4). On sagittal views, they resemble a string of beads. The thyroid gland (with a speckled, homogeneous, hyperechoic appearance) and the isthmus should then be delineated at the level of the suprasternal notch (Fig. 5).

Transverse/axial real-time imaging of the trachea allows continuous visualisation of the tracheal midline. Deviation of the guide wire too far from the midline can occur and can cause paratracheal tissue damage during the subsequent dilatation. Placing the transducer transverse to the tracheal cartilage and first obtaining a short-axis US image allow the identification of the tracheal midline, thereby helping to avoid this problem. However, on this view, it can be difficult to identify the correct space between the tracheal rings and to differentiate cartilage from air. We therefore recommend obtaining the details of the midline of the neck on the transverse/axial view, determining the absence of aberrant structures, and then moving the probe 90 degrees to obtain a longitudinal view with the cricoid cartilage and the first tracheal ring in the centre of the US screen. On this view, the space between the first and second or second and third tracheal cartilage at the midline of the trachea can be identified (Fig. 4). This approach allows the needle, a 15-G thin-wall needle, to be followed as it is advanced while the laryngeal cartilage and the tracheal cartilage are visible and can be easily differentiated from the air between the cartilages. As the needle passes through the anterior wall, a change in resistance is felt and the lumen is entered, followed by the aspiration of air/fluid into the attached syringe.

This so-called in-plane approach can sometimes be difficult in patients with short necks because of a lack of physical space. In such cases, a perpendicular angle of puncture is preferred, despite the increased risk of injury to the posterior wall of the trachea. In this approach, the transverse probe is positioned immediately above the level of the planned puncture. With slight caudal angulation of the probe, good visualisation and direction of the needle tip toward the tracheal midline should be possible. The needle is introduced perpendicularly to the skin, and the path is determined by the distinct acoustic shadow ahead of the needle, followed by the displacement of tissue layers observed with needle passage. The indentation of the anterior tracheal wall by the needle can sometimes be observed on these US images. This technique has been described by Chacko and colleagues [31].

As soon as a loss of resistance is felt or air or fluid is aspirated into the attached syringe, the US probe is set aside, and the syringe is disconnected. A guide wire from a pre-assembled tracheostomy kit is then inserted through the needle into the trachea as a guide for further tracheostomy. Finally, a bronchoscope is used to determine whether injury to the posterior wall of the trachea has occurred.

## Conclusions

As US technology continues to evolve, its scope is becoming limited only by operator expertise and not by the capabilities of the bedside unit. USS of the upper airway can provide a great deal of anatomical information that would not be evident with clinical examination alone. With the development of better probes, high-resolution imaging, real-time pictures, and clinical experience, USS has become a potential first-line noninvasive airway assessment tool in intensive care practice. However, until prospective RCTs are conducted to evaluate the safety and efficacy of US-PDT compared with the traditional landmark-guided technique, the best available evidence highly recommends the use of USS prior to, during, and after PDT to improve the safety of the procedure.

## Abbreviations

A-M: Air-mucosal
PDT: Percutaneous dilatational tracheostomy
RCT: Randomised controlled trial
US: Ultrasound
US-PDT: Ultrasound-guided percutaneous dilatational tracheostomy
USS: Ultrasound scanning
